# Sex-specific co-occurrence patterns of Type 2 Diabetes Mellitus and Non-Alcoholic Fatty Liver Disease among patients with colorectal cancer: a retrospective EMR-based series

**DOI:** 10.3389/fmed.2026.1736652

**Published:** 2026-07-02

**Authors:** Mohammadreza Akbarian Khorasgani, Pouriya Katouzi, Melika Khalifeh Hadi, Masoumeh Amarloei, Yasaman Kianpourhafshejani, Sumaeya Sultana, Mohammed Hasan Mohammed Ahmed Al-Mlawi, Yunqing Zeng, Jiaoyang Lu

**Affiliations:** 1 Department of Gastroenterology, Qilu Hospital of Shandong University, Jinan, China; 2 Shandong University Cheeloo College of Medicine, Jinan, China; 3 The Second Clinical Medical School of Shandong University, Jinan, China

**Keywords:** colorectal cancer, co-occurrence, EMR analysis, metabolic comorbidities, Non-Alcoholic Fatty Liver Disease, retrospective study, sex differences, Type 2 Diabetes Mellitus

## Abstract

**Background:**

Colorectal cancer (CRC) is a major global health burden and one of the most prevalent malignancies worldwide. Its association with metabolic comorbidities is receiving increasing attention. Type 2 Diabetes Mellitus (T2DM) and Non-Alcoholic Fatty Liver Disease (NAFLD) are two interrelated metabolic disorders increasingly implicated in CRC pathogenesis, possibly via insulin resistance, chronic inflammation, oxidative stress, and gut–liver axis dysregulation. However, limited evidence exists on their co-occurrence and sex-specific distribution among CRC patients, particularly within real-world clinical settings in Asian populations. This study explored the sex-specific prevalence and co-occurrence patterns of T2DM and NAFLD among CRC patients and evaluated associated metabolic profiles using electronic medical records (EMRs).

**Methods:**

We conducted a retrospective EMR-based series study involving 438 CRC patients treated at a tertiary hospital in China, all of whom met strict inclusion criteria, including a complete diagnostic history and colonoscopy between January 2020 and December 2024. Diagnoses of T2DM and NAFLD were confirmed based on explicit physician-documented records. Descriptive statistics and bivariate analyses (chi-square/Fisher’s exact and appropriate parametric or non-parametric tests) were used to evaluate prevalence patterns and metabolic indicators.

**Results:**

T2DM and NAFLD were more prevalent in male CRC patients (10.04 and 8.18%, respectively) than in females (5.92 and 4.73%). However, the co-occurrence of both conditions was rare (0.68%). Patients with T2DM or NAFLD showed distinctive metabolic abnormalities, including elevated blood sugar, liver enzymes, and altered lipid profiles. Bivariate analysis identified AST as a potential differentiating marker for NAFLD. Because co-occurrence was rare (3/438, 0.68%), exact analysis showed no evidence of a sex difference in co-occurrence (male vs. female OR = 1.26, 95% CI 0.11–13.99; *p* = 1.00).

**Conclusion:**

This study highlights distinct sex-based prevalence patterns of T2DM and NAFLD in CRC patients; however, co-occurrence was rare, limiting inferential analyses. These findings emphasize the need for larger, prospective studies with refined ascertainment to better characterize metabolic comorbidity patterns in CRC, particularly from a sex-specific perspective.

## 1 Introduction

Colorectal cancer (CRC) remains one of the leading malignancies worldwide, responsible for significant morbidity and mortality, with approximately 1.9 million new cases and 935,000 deaths annually (1). The rising incidence of CRC is particularly notable in regions experiencing rapid changes in lifestyle, dietary patterns, and metabolic health.

Metabolic disorders, particularly Type 2 Diabetes Mellitus (T2DM) and Non-Alcoholic Fatty Liver Disease (NAFLD), have been individually recognized as significant contributors to CRC risk. Robust epidemiological evidence consistently demonstrates that T2DM independently increases CRC risk by approximately 20–30% (2), primarily through mechanisms involving insulin resistance, chronic low-grade inflammation, hyperinsulinemia, oxidative stress, and gut-liver axis disturbances (3). NAFLD similarly poses an elevated risk for colorectal adenomas and cancer, especially in cases characterized by advanced hepatic fibrosis or non-alcoholic steatohepatitis (NASH) (4).

While the independent associations of T2DM and NAFLD with CRC have been well documented, limited research has described their co-occurrence patterns among CRC patients, particularly when considering sex-specific differences. Biological variations between sexes, such as hormonal regulation, visceral adiposity distribution, and immune response modulation, may substantially affect the interplay of these metabolic conditions and their association with CRC (5). Nonetheless, existing studies have inadequately explored or reported these sex-specific interactions, leaving a critical knowledge gap.

To address this gap, this retrospective electronic medical record (EMR)-based study conducted at a high-volume tertiary care institution investigates sex-specific co-occurrence patterns of T2DM and NAFLD among CRC patients. By leveraging real-world clinical data, our study aims to describe whether and how the presence of these metabolic comorbidities differs between male and female CRC patients and to characterize associated metabolic profiles. This approach may enhance understanding of comorbidity patterns and inform future hypothesis-driven studies.

In this context, the term “co-occurrence patterns” refers solely to the descriptive presence of both T2DM and NAFLD within the CRC patient population and does not imply causality or risk modification.

## 2 Materials and methods

### 2.1 Study design and setting

This retrospective EMR-based series study utilized de-identified electronic medical records (EMRs) from Qilu Hospital, a high-volume tertiary hospital affiliated with Shandong University. Data from adult patients (aged ≥16 years) who underwent colonoscopy and had complete clinical documentation between January 2020 and December 2024 were initially included. Although a total of 500 patients met general inclusion criteria, only 438 fulfilled all inclusion and exclusion requirements based on colonoscopy timing and data completeness.

This study was reviewed and approved by the Ethics Committee of Qilu Hospital of Shandong University (approval number KYLL-202509-058). The committee determined that the research met ethical standards for retrospective studies using de-identified medical record data and granted a waiver of informed consent. All data were anonymized prior to analysis, with no direct patient contact or access to identifiable information. All procedures followed the Declaration of Helsinki and institutional ethical guidelines.

### 2.2 Study population and eligibility criteria

Patients included in the study had colonoscopically and histologically confirmed colorectal cancer (CRC). Inclusion criteria required complete demographic and anthropometric data and sufficient clinical documentation for comorbidity ascertainment (T2DM/NAFLD). Diagnostic criteria were strictly defined:

- T2DM was recorded as present only when explicitly diagnosed in past medical history or current diagnosis sections of EMRs, irrespective of laboratory glucose measurements at data extraction.
- NAFLD was similarly classified based on explicit EMR documentation, typically involving ultrasound imaging confirmation or physician-documented diagnosis. Elevated liver enzymes (ALT, AST) or abnormal FIB-4 scores alone, without explicit clinical documentation, did not suffice for NAFLD diagnosis.

In routine clinical practice, NAFLD documentation in EMRs is primarily based on clinically indicated imaging impressions and physician diagnoses rather than protocolized screening; therefore, NAFLD ascertainment in this study reflects recorded clinical diagnoses. Accordingly, some mild or unrecorded steatosis may not be captured in EMR-based case identification.

This strategy was adopted to ensure high diagnostic specificity and minimize misclassification due to transient laboratory abnormalities or incomplete data. We acknowledge that this approach may underestimate the true prevalence of subclinical or undocumented cases, particularly for NAFLD, but believe it provides a reliable and reproducible method consistent with real-world clinical data recording (6).

In addition, patients were excluded if they had inflammatory bowel disease (IBD; e.g., Crohn’s disease, ulcerative colitis), hereditary colorectal cancer syndromes (e.g., Lynch syndrome, familial adenomatous polyposis), or incomplete data critical for analysis.

### 2.3 Variables and definitions

The primary outcomes included sex-specific prevalence and co-occurrence distribution patterns of T2DM and NAFLD among CRC patients. Secondary variables examined included demographic details (age, sex), anthropometric data (Body Mass Index [BMI]), clinical comorbidities (hypertension), lifestyle characteristics (smoking status, alcohol use), and laboratory markers (blood glucose levels [BS], triglycerides [TG], Low-Density Lipoprotein [LDL], High-Density Lipoprotein [HDL]). Liver-related parameters (ALT, AST, FIB-4 scores) were included to characterize hepatic profiles.

### 2.4 Data collection and processing

Data were manually extracted from EMRs and systematically entered into Excel spreadsheets using standardized formats, with dropdown menus ensuring accuracy. Quality control involved two independent reviewers checking for duplicates, data consistency, and resolving any discrepancies. Extreme laboratory values were flagged during data extraction. Each flagged value was manually cross-checked against the original EMR to verify accuracy and rule out entry errors. Verified values were retained as true clinical findings. Standardized formulae were uniformly applied for BMI and FIB-4 calculations.

### 2.5 Statistical analysis

Descriptive statistics summarized patient demographics and clinical data. Chi-square tests evaluated relationships between categorical variables. Given the extremely low frequency of T2DM–NAFLD co-occurrence, analyses were primarily descriptive and based on exact/bivariate methods for categorical comparisons. All statistical analyses were performed using SPSS version 27.0.1, with statistical significance set at a two-tailed *p*-value <0.05.

For bivariate analyses of categorical variables, we initially employed the chi-square test. However, due to small expected frequencies in several contingency table cells—particularly in subgroups with rare co-occurrence—we supplemented this with Fisher’s exact test, which provides more accurate significance estimates under such conditions (7). This dual approach allowed for a more robust interpretation of categorical associations, minimizing the risk of Type I and Type II errors associated with low cell counts.

To clarify, we use the term “co-occurrence” to denote descriptive presence of both T2DM and NAFLD among CRC patients, without implying statistical interaction or CRC risk inference.

### 2.6 Study timeline and resources

This study spanned approximately 3 months, encompassing data extraction, statistical analysis, and manuscript preparation. Resources utilized included hospital EMRs, Excel software for data management, and SPSS software for statistical analyses, requiring an estimated weekly commitment of 20 h per research team member.

## 3 Results

### 3.1 Descriptive statistics

#### 3.1.1 Patient demographics and clinical characteristics

The final study population included 438 patients who met all inclusion and exclusion criteria within the specified colonoscopy date range. The average age and Body Mass Index (BMI) differed slightly by sex, with males being older and having higher BMI on average (Table 1).

**Table 1:** Age & BMI by sex.

Sex	Age (mean)	Age (SD)	BMI (mean)	BMI (SD)
Female	56.95	13.51	22.91	3.46
Male	61.05	10.61	24.39	3.37

Males had a slightly higher average age and BMI compared to females, suggesting a modest difference in risk profile distribution by sex.

#### 3.1.2 CRC location and histology distribution

The sigmoid colon was the most common CRC tumor location in both sexes. However, notable sex-specific differences were observed: rectal cancer was more frequent in males, while ascending colon cancer was more common in females (Table 2). Adenocarcinoma was the predominant histological type in both sexes, with a rare occurrence of squamous cell carcinoma in females (Table 3).

**Table 2:** CRC location distribution by sex.

CRC location	Female (%)	Male (%)
Ascending colon	23.08	13.75
Colon (unspecified)	24.85	23.42
Descending colon	3.55	6.32
Rectum	4.73	9.29
Sigmoid	37.28	40.15
Transverse colon	6.51	7.06

Sigmoid colon was the most common tumor location in both sexes, 37% in females and 40% in males. However, rectum cancer was nearly twice as common in males at 9.29% compared to 4.73% in females. Similarly, ascending colon involvement was more frequent in females at 23.08% than in males at 13.75%. This suggests possible differences in anatomical or pathological distribution patterns in colorectal cancer based on sex.

**Table 3:** CRC histological type distribution by sex.

CRC histology	Female (%)	Male (%)
Adenocarcinoma	99.41	100.0
Squamous cell carcinoma	0.59	0.0

Adenocarcinoma was the predominant histological type in both sexes, with only a rare case of squamous cell carcinoma observed in females.

### 3.2 Prevalence rates

#### 3.2.1 Prevalence of T2DM and NAFLD

**Figure 1:**
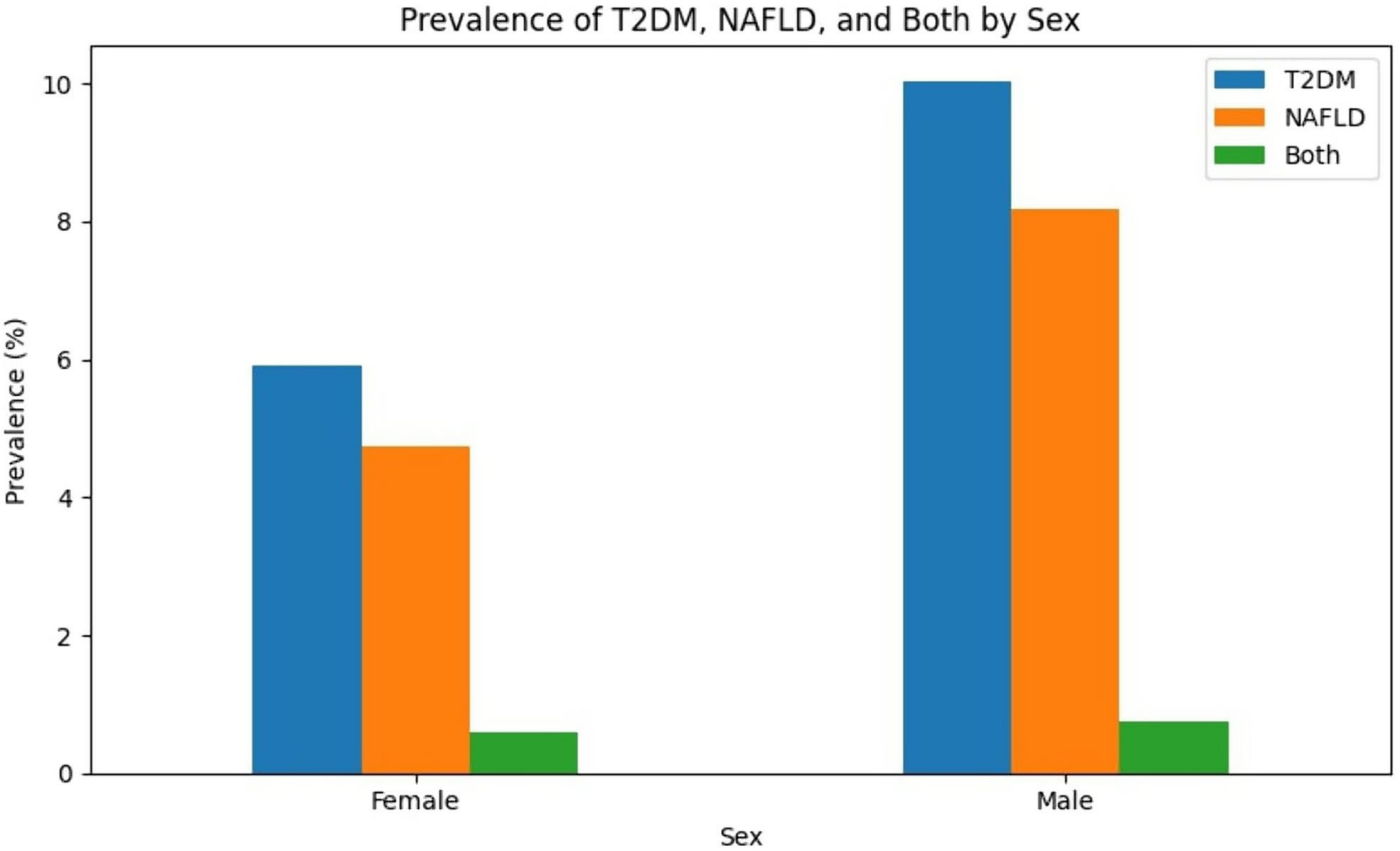
Prevalence of T2DM, NAFLD, and both by sex. Co-occurrence of T2DM and NAFLD was rare (0.68% overall), and slightly higher in males. Males also had a higher proportion of both individual conditions.

The prevalence of Type 2 Diabetes Mellitus (T2DM) was higher in males compared to females (10.04% vs. 5.92%, respectively) (Supplementary Table S1) (Table 4). Similarly, Non-Alcoholic Fatty Liver Disease (NAFLD) was more prevalent among males (8.18%) than females (4.73%) (Supplementary Table S2) (Table 5). The overall co-occurrence of T2DM and NAFLD was rare (0.68%), slightly higher among males (Supplementary Table S3) (Table 6; Figure 1).

**Table 4:** Prevalence of T2DM by sex.

Sex	T2DM: no (%)	T2DM: yes (%)
Female	94.08	5.92
Male	89.96	10.04

T2DM was more prevalent among males (10.04%) compared to females (5.92%).

**Table 5:** Prevalence of NAFLD by sex.

Sex	NAFLD: no (%)	NAFLD: yes (%)
Female	95.27	4.73
Male	91.82	8.18

NAFLD was also more frequent in males than females, showing a sex-specific difference in prevalence.

**Table 6:** Co-occurrence of T2DM & NAFLD by sex.

Sex	Both (%)	NAFLD only (%)	Neither (%)	T2DM only (%)
Female	0.59	4.14	89.94	5.33
Male	0.74	7.43	82.53	9.29

### 3.3 Metabolic marker analysis

Patients with T2DM exhibited elevated blood sugar (BS) and FIB-4 scores, alongside lower HDL and LDL cholesterol levels, reflecting a more adverse metabolic profile (Supplementary Table S4). NAFLD patients demonstrated increased ALT, AST, and triglyceride (TG) levels, indicative of hepatic metabolic stress (Supplementary Table S5) (8). Females exhibited significantly higher HDL and liver enzyme levels (ALT, AST), whereas males had higher BS and triglyceride levels (Supplementary Table S6; Supplementary Figures S1–S3).

### 3.4 Subpopulation analysis

Older patients (>50 years) showed higher prevalence rates for T2DM, NAFLD, and notably hypertension (Supplementary Tables S7–S9). Obesity (BMI ≥ 30 kg/m^2^) was associated with slightly higher T2DM and hypertension prevalence but not significantly higher NAFLD prevalence (Supplementary Table S10).

### 3.5 Bivariate comparisons

#### 3.5.1 Comparative analysis using chi-square, Fisher’s exact, independent *t*-tests, and Mann–Whitney U tests

**Figure 2:**
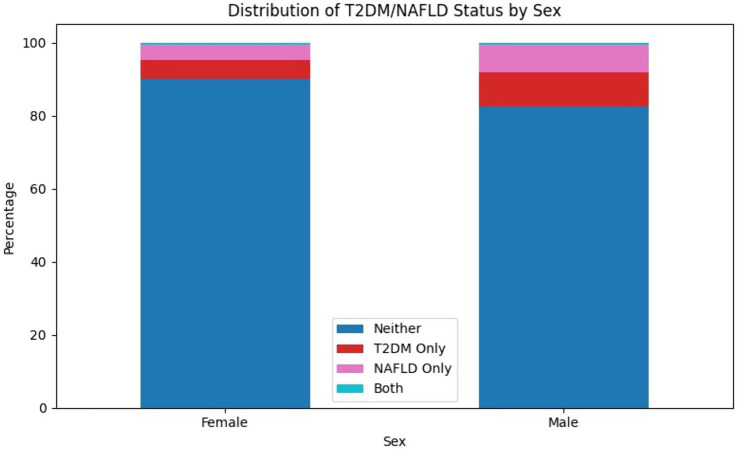
Distribution of T2DM & NAFLD by sex.

Chi-square and Fisher’s exact tests revealed no significant association between T2DM and NAFLD, nor between these conditions and sex (Supplementary Tables S11–S14) (Figure 2). Independent *t*-tests and Mann–Whitney U tests identified AST levels as significantly elevated in NAFLD patients, highlighting AST as a potential key differentiating marker (Table 7). ALT and FIB-4 comparisons are shown in Supplementary Tables S15, S16.

**Table 7:** Comparative analysis of AST level by T2DM, NAFLD, and co-occurrence status using independent *t*-tests and Mann–Whitney U tests.

Group comparison	*p*-value	Effect size
T2DM: yes vs. no	0.4302	0.125
NAFLD: yes vs. no	0.0023	0.312
T2DM + NAFLD: both vs. none	0.5980	0.068

Because T2DM–NAFLD co-occurrence was rare (3/438, 0.68%), sex differences were evaluated using Fisher’s exact test. There was no evidence of a sex difference in co-occurrence (male vs. female OR = 1.26, 95% CI 0.11–13.99; *p* = 1.00; Supplementary Table S14).

## 4 Discussion

This retrospective EMR-based series study evaluated the sex-specific co-occurrence patterns of Type 2 Diabetes Mellitus (T2DM) and Non-Alcoholic Fatty Liver Disease (NAFLD) among patients diagnosed with colorectal cancer (CRC). Our analysis included 438 patients meeting stringent inclusion criteria, demonstrating distinct sex-based patterns in CRC location and metabolic characteristics.

Our findings indicated notable differences in CRC localization by sex, with males more commonly presenting with rectal cancer and females showing higher prevalence in the ascending colon. These differences might be attributed to hormonal, genetic, and lifestyle factors influencing tumorigenesis in distinct colon segments, a phenomenon documented in previous epidemiological studies. Such sex-specific variations underscore the importance of targeted screening and management strategies in CRC patients.

Importantly, the phrase “co-occurrence patterns” is used in this study to describe observational co-occurrence rates between T2DM and NAFLD among CRC patients, and is not intended to imply mechanistic synergy or causal interaction influencing CRC development.

The observed prevalence rates for T2DM and NAFLD were higher in male CRC patients compared to females. This aligns with prior literature suggesting sex-related disparities in metabolic syndrome components, likely influenced by differential adiposity distribution, hormonal regulation, and lifestyle risk factors. Despite this, the overall co-occurrence of T2DM and NAFLD was very rare, limiting our capacity to statistically validate their potential combined comorbidity impact.

Metabolic marker analysis revealed distinct profiles for T2DM and NAFLD groups. Patients with T2DM exhibited elevated blood glucose levels and higher FIB-4 scores indicative of advanced liver fibrosis risk, which may correlate with chronic hyperglycemia and insulin resistance exacerbating hepatic dysfunction. Conversely, NAFLD patients showed elevated hepatic enzymes (ALT and AST) and triglyceride levels, confirming hepatic lipid accumulation and associated inflammation as primary characteristics. Interestingly, females displayed significantly higher HDL cholesterol and hepatic enzyme levels, whereas males exhibited higher glucose and triglyceride concentrations, highlighting sex-specific metabolic responses that may influence disease progression and outcomes (9).

Subpopulation analyses revealed that older age and obesity were associated with higher prevalence of metabolic conditions, consistent with existing evidence linking age-related metabolic dysregulation and adiposity-driven inflammation to increased CRC risk. Nonetheless, obesity did not significantly elevate NAFLD prevalence in our study, possibly due to strict clinical diagnostic criteria rather than surrogate biomarkers alone.

Bivariate analyses identified AST as significantly elevated in NAFLD patients, consistent with previous reports suggesting AST as a marker of hepatic inflammation and fibrosis. Because T2DM–NAFLD co-occurrence was rare, inferential analyses of predictors of co-occurrence were limited and exact testing did not show evidence of a sex difference in co-occurrence.

Limitations of our study include retrospective data reliance, potential underdiagnosis due to stringent clinical diagnostic criteria, and limited sample size for co-occurrence analysis. Moreover, the retrospective design introduces inherent limitations such as misclassification bias, particularly due to variation in EMR documentation and diagnostic workup driven by clinical indication. In addition, heterogeneity in imaging modality and radiology reporting language over time may have contributed to under-ascertainment of NAFLD. Patients with subclinical or undocumented disease may have been misclassified despite our strict inclusion criteria. Accordingly, our EMR documentation-based definitions likely introduced a false-negative rate for both T2DM and NAFLD, which may have underestimated their true prevalence and co-occurrence. Therefore, any assessment of statistical interaction or “synergy” between T2DM and NAFLD is not supported in this dataset and our findings should be interpreted as descriptive co-occurrence patterns. Temporality also poses a challenge in interpreting causality; we could not determine whether T2DM or NAFLD preceded CRC onset or vice versa. Additionally, residual confounding may exist due to unmeasured factors such as dietary patterns, physical activity, medication adherence, and genetic predispositions that were not available in the EMRs. Information on specific metabolic medications (e.g., metformin, statins) and CRC stage was not uniformly available for structured analysis across all records and was therefore not included as covariates. Future studies employing prospective designs, broader diagnostic criteria, and larger, more diverse populations are essential to better delineate the relationship between metabolic diseases and CRC, especially regarding combined comorbidity impacts.

Ultimately, this study highlights distinct sex-specific metabolic and pathological patterns among CRC patients, emphasizing the necessity for personalized screening strategies. Although the anticipated co-occurrence prevalence of T2DM and NAFLD was rare, this finding underscores the complexity of metabolic disease interactions and the importance of larger-scale investigations to adequately assess their collective impact on colorectal cancer.

## 5 Conclusion

This retrospective EMR-based series study identified sex-specific patterns in the prevalence of Type 2 Diabetes Mellitus (T2DM) and Non-Alcoholic Fatty Liver Disease (NAFLD) among patients with colorectal cancer (CRC). We observed that both T2DM and NAFLD were more prevalent among male CRC patients. However, the co-occurrence of these conditions remained exceptionally rare across both sexes. Although distinct metabolic profiles—including elevated liver enzymes and lipid abnormalities—were present in patients with either condition, because T2DM–NAFLD co-occurrence was rare, interaction (“synergy”) inference was not supported and analyses were interpreted descriptively, including exact testing for sex differences in co-occurrence. This limitation reflects the low frequency of T2DM–NAFLD co-occurrence and limited statistical power for subgroup inference. These findings emphasize the need for larger, prospective cohort studies to better elucidate potential co-occurrent interactions and sex-specific implications of these metabolic disorders in CRC. Future research should prioritize improved diagnostic accuracy and integrated metabolic profiling to untangle these complex interrelationships.

## Data Availability

The raw data supporting the conclusions of this article will be made available by the authors, without undue reservation.
