# Supplementary material for: Sex-specific co-occurrence patterns of Type 2 Diabetes Mellitus and Non-Alcoholic Fatty Liver Disease among patients with colorectal cancer: a retrospective EMR-based series
Source: Front Med (Lausanne). 2026 Jul 2;13:1736652. doi: 10.3389/fmed.2026.1736652 (PMC13374828; doi:10.3389/fmed.2026.1736652)

Supplementary Material

1 Supplementary Tables

Table S1. Prevalence of T2DM.

T2DM Status	Count	Percent (%)
**No**	401	91.55
**Yes**	37	8.45

Table S2. Prevalence of NAFLD.

NAFLD Status	Count	Percent (%)
**No**	408	93.15
**Yes**	30	6.85

Table S3. Co-occurrence of T2DM & NAFLD.

Condition	Percent (%)
**Neither**	85.39
**T2DM Only**	7.76
**NAFLD Only**	6.16
**Both (Co-occurring)**	0.68

Table S4. Marker Summary by T2DM Status.

T2DM	ALT (mean)	ALT (median)	AST (mean)	AST (median)	FIB-4 (mean)	FIB-4 (median)	BS (mean)	BS (median)	HDL (mean)	HDL (median)	LDL (mean)	LDL (median)	TG (mean)	TG (median)
**No**	22.78	16.0	27.22	20.0	1.83	1.42	6.37	5.04	1.62	1.25	3.33	2.82	1.48	1.18
**Yes**	19.78	16.0	20.16	16.0	2.55	1.56	7.05	6.71	1.27	1.22	2.58	2.35	1.49	1.33

T2DM patients had higher FIB-4 and BS levels, and lower HDL and LDL values, consistent with a more adverse metabolic profile.

Table S5. Marker Summary by NAFLD Status.

NAFLD	ALT (mean)	ALT (median)	AST (mean)	AST (median)	FIB-4 (mean)	FIB-4 (median)	BS (mean)	BS (median)	HDL (mean)	HDL (median)	LDL (mean)	LDL (median)	TG (mean)	TG (median)
No	22.31	16.0	26.40	20.0	1.88	1.43	6.51	5.13	1.61	1.24	3.30	2.79	1.45	1.18
Yes	25.53	20.0	29.60	22.0	1.97	1.30	5.38	5.13	1.31	1.34	2.78	2.87	1.85	1.41

NAFLD patients showed elevated ALT, AST, and TG levels, along with reduced HDL — characteristic features of hepatic metabolic stress.

Table S6. Marker Summary by Sex.

Sex	ALT (mean)	ALT (median)	AST (mean)	AST (median)	FIB-4 (mean)	FIB-4 (median)	BS (mean)	BS (median)	HDL (mean)	HDL (median)	LDL (mean)	LDL (median)	TG (mean)	TG (median)
Female	26.24	18.0	32.59	22.0	1.95	1.40	5.37	5.10	2.18	1.36	4.02	2.85	1.42	1.22
Male	20.20	16.0	22.88	18.0	1.86	1.42	7.10	5.13	1.22	1.18	2.79	2.75	1.52	1.19

Females exhibited significantly higher HDL and liver enzyme levels, while males had higher BS and TG values, reflecting distinct metabolic profiles by sex.

Table S7. T2DM Prevalence by Age Group.

Age Group	T2DM: No (%)	T2DM: Yes (%)
**≤50**	93.75	6.25
**>50**	90.94	9.06

Older patients (>50) had a higher prevalence of T2DM compared to younger patients.

Table S8. NAFLD Prevalence by Age Group.

Age Group	NAFLD: No (%)	NAFLD: Yes (%)
**≤50**	94.79	5.21
**>50**	92.69	7.31

NAFLD prevalence was modestly higher in older patients.

Table S9. Hypertension Prevalence by Age Group.

Age Group	Hypertension: No (%)	Hypertension: Yes (%)
**≤50**	90.62	9.38
**>50**	67.54	32.46

There was a substantial increase in hypertension prevalence among older patients, with nearly 1 in 3 showing elevated blood pressure.

Table S10. T2DM, NAFLD & Hypertension Prevalence by Obesity Status.

Obesity Group	T2DM: No (%)	T2DM: Yes (%)	NAFLD: No (%)	NAFLD: Yes (%)	HTN: No (%)	HTN: Yes (%)
**Non-Obese**	91.63	8.37	93.06	6.94	72.73	27.27
**Obese (BMI ≥ 30)**	90.00	10.00	95.00	5.00	70.00	30.00

Among the study population, 20 patients (4.57%) were classified as obese (BMI ≥ 30), while 418 patients (95.43%) were non-obese (BMI < 30). The sample size for obese individuals is notably small. T2DM and hypertension were slightly more prevalent in the obese group, whereas NAFLD appeared marginally less common. However, these differences were not substantial.

Table S11. Chi-Square Analysis of the Association Between T2DM & NAFLD.

	NAFLD: No	NAFLD: Yes
**T2DM: No**	428	29
**T2DM: Yes**	40	3

Chi-square p-value is 1.00. No significant association was found between T2DM and NAFLD

Table S12. Chi-Square Analysis of the Association Between T2DM & Sex.

	Female	Male
**T2DM: No**	177	280
**T2DM: Yes**	11	32

Chi-square p-value is 0.1242. Slightly higher T2DM prevalence in males, but not statistically significant.

Table S13. Chi-Square Analysis of the Association Between NAFLD & Sex.

	Female	Male
**NAFLD: No**	180	288
**NAFLD: Yes**	8	24

Chi-square p-value is 0.1827. NAFLD also more common in males, but not statistically significant.

Table S14. Fisher's Exact Test of T2DM and NAFLD Co-occurrence by Sex.

Group	Co-occurrence = No	Co-occurrence = Yes
**Female**	168	1
**Male**	267	2

Fisher's exact test comparing co-occurrence between males and females: OR (male vs female) =1.26, 95% CI 0.11–13.99, p=1.00. Co-occurrence defined as presence of both T2DM and NAFLD.

Table S15. Comparative Analysis of ALT Level by T2DM, NAFLD, and Co-occurrence Status Using Independent t-tests and Mann–Whitney U Tests.

Group Comparison	p-value	Effect Size
**T2DM: Yes vs No**	0.3597	0.145
**NAFLD: Yes vs No**	0.2838	0.140
**T2DM+NAFLD: Both vs None**	0.6224	0.069

Table S16. Comparative Analysis of FIB-4 Score by T2DM, NAFLD, and Co-occurrence Status Using Independent t-tests and Mann–Whitney U Tests.

Group Comparison	p-value	Effect Size
**T2DM: Yes vs No**	0.8042	0.043
**NAFLD: Yes vs No**	0.4389	0.177
**T2DM+NAFLD: Both vs None**	0.8377	0.051

2 Supplementary Figures

Fig S1. Comparison of Serum ALT Levels Stratified by T2DM & NAFLD.

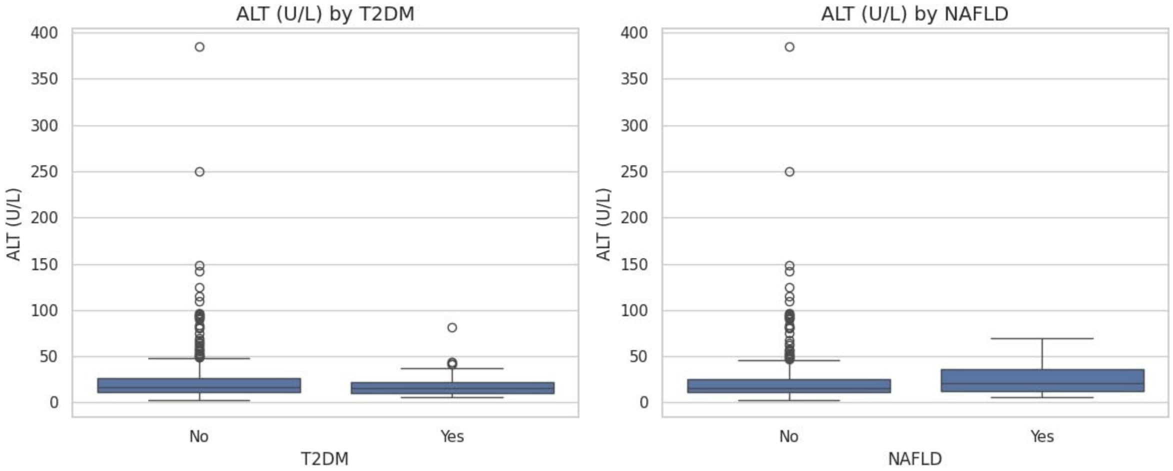

Fig S2. Comparison of Serum AST Levels Stratified by T2DM & NAFLD.

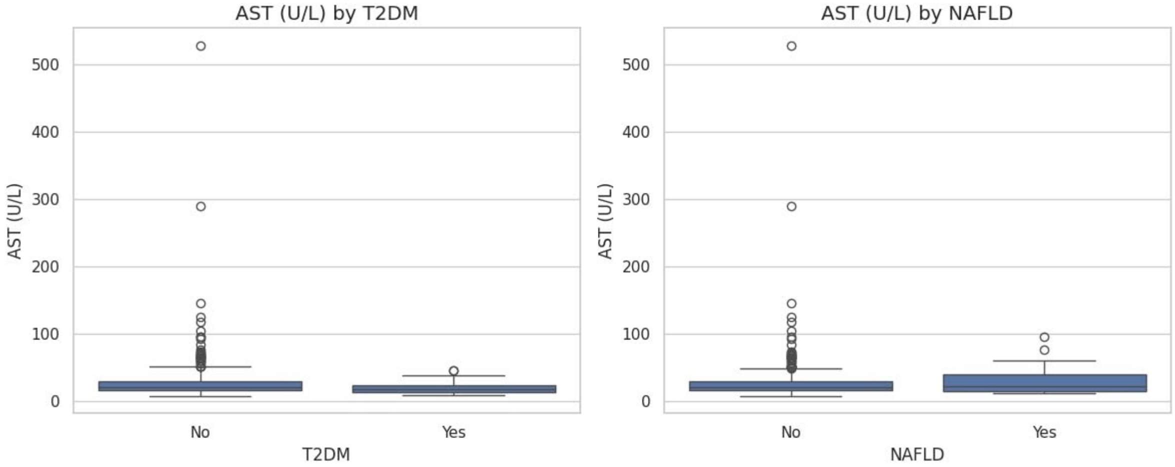

Fig S3. Comparison of FIB-4 Score Stratified by T2DM & NAFLD.

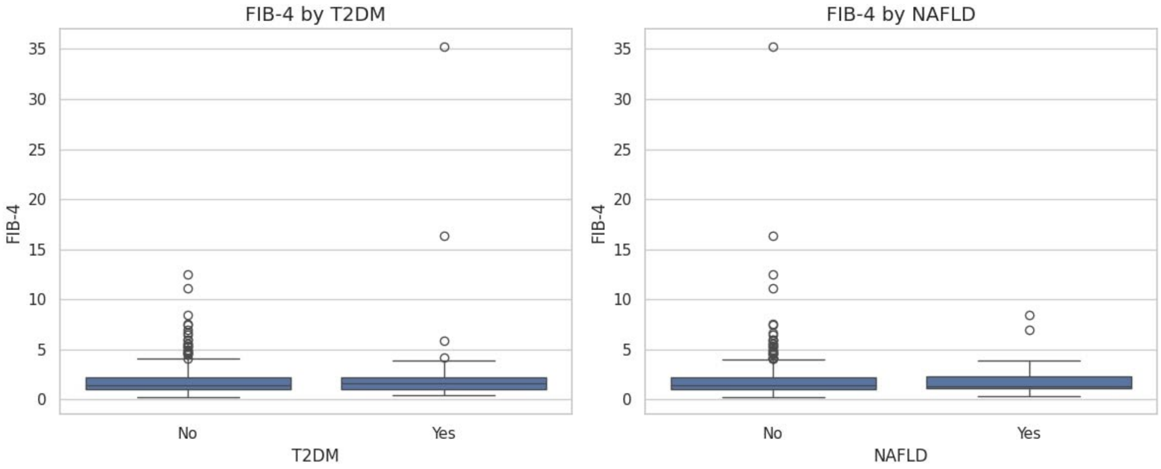

Supplement: Supplementary file 1 [file Data_Sheet_1.pdf]
